# The Axillary Nodal Harvest in Breast Cancer Surgery Is Unchanged by Sentinel Node Biopsy or the Timing of Surgery

**DOI:** 10.1155/2012/467825

**Published:** 2012-05-30

**Authors:** B. E. Byrne, R. I. Cutress, J. Gill, M. H. Wise, C. Yiangou, A. Agrawal

**Affiliations:** ^1^Department of General Surgery, Royal Bournemouth Hospital, Royal Bournemouth and Christchurch NHS Foundation Trust, Castle Lane East, Bournemouth BH7 7DW, UK; ^2^Southampton Breast Unit, Princess Anne Hospital, Southampton University Hospitals Trust, C Level Mailpoint 132, Coxford Road, Southampton SO16 5YA, UK; ^3^Breast Unit, Musgrove Park Hospital, Taunton & Somerset NHS Foundation Trust, Taunton, Somerset TA1 5DA, UK; ^4^Breast Care Centre, Queen Alexandra Hospital, Portsmouth Hospitals NHS Trust, Portsmouth PO6 3LY, UK

## Abstract

*Introduction*. Patients with a positive sentinel lymph node biopsy may undergo delayed completion axillary dissection. Where intraoperative analysis is available, immediate completion axillary dissection can be performed. Alternatively, patients may undergo primary axillary dissection for breast cancer, historically or when preoperative assessment suggests axillary metastases. This study aims to determine if there is a difference in the total number of lymph nodes or the number of metastatic nodes harvested between the 3 possible approaches. *Methods*. Three consecutive comparable groups of 50 consecutive patients who underwent axillary dissection in each of the above contexts were identified from the Portsmouth Breast Unit Database. Patient demographics, clinicopathological variables, and surgical treatment were recorded. The total pathological nodal count and the number of metastatic nodes were compared between the groups. *Results*. There were no differences in clinico-pathological features between the three groups for all features studied with the exception of breast surgical procedure (*P* < 0.001). There were no differences in total nodal harvest (*P* = 0.822) or in the number of positive nodes harvested (*P* = 0.157) between the three groups. *Conclusion*. The three approaches to axillary clearance yield equivalent nodal harvests, suggesting oncological equivalence and robustness of surgical technique.

## 1. Introduction

The role of axillary surgery in breast cancer is to stage the axilla and in those with lymph node metastases to treat the axilla with axillary clearance [1]. Adequate axillary dissection is important in node-positive patients both to ensure removal of all involved nodes to optimise local control and to obtain the maximum prognostic information [2]. When staging the axilla, an additional goal, particularly in node-negative patients, is to minimise morbidity. Various strategies for doing so have been developed, the most recent being dual localisation sentinel lymph node biopsy (SLNB). This has been recommended by the United Kingdom National Institute for Health and Clinical Excellence as the “preferred technique” for staging the axilla in radiologically or cytologically (where tested) node-negative patients [3]. If SLNB analysis demonstrates metastasis to the axilla, it is recommended that patients undergo axillary clearance [4].

Traditionally, sentinel lymph nodes are analysed histologically, and patients who are found to have metastases in these nodes often undergo a delayed completion axillary dissection (dALND) after a delay when the histological result is available. In two large United Kingdom datasets of SLNB, the ALMANAC trial [5] and the New Start training programme [6], approximately 30% of patients were SLNB positive and therefore required further axillary surgery. Alternative analysis techniques have been developed for use in conjunction with SLNB to enable rapid intraoperative analysis of sentinel lymph nodes including touch imprint cytology, frozen section analysis, and molecular analysis such as OSNA and the Veridex polymerase chain reaction (PCR) assay as used in our institution. These diagnostic techniques enable node-positive patients to undergo immediate axillary clearance under the same general anaesthetic (GA) without the need for a second operation and another GA.

Anecdotally, dALND is often more technically challenging than primary axillary clearance or immediate completion axillary dissection (iALND) following intraoperative SLNB analysis. This is supported by data from Goyal et al., who have shown that a dALND requires a greater operative time than iALND [7]. With increased difficulty associated with delayed axillary clearance, it is important to establish whether optimal oncological results are maintained using this approach. It is also important to establish oncological equivalence between primary axillary clearance and SLNB with iALND, as although there is no scarring to contend with, the axillary contents are fragmented by the latter approach. Chakravorty et al. have shown no difference in median nodal yields from axillary clearance with or without SLNB, in either the immediate or delayed setting [8]. Earlier studies support these findings, but only compared two of the three possible patient groups [7, 9].

We determined to assess the nodal harvest from the axilla in these various contexts, within a single centre, with the same surgeons and pathologists all working in accordance with the same standardised protocols. Our hypothesis was that dALND might yield a lower number of lymph nodes due to increased technical difficulty and scarring compared to iALND.

## 2. Methods

### 2.1. Techniques

Sentinel node biopsy using dual localisation with radioisotope and Patent Blue V dye was introduced into Portsmouth Breast Unit for staging the axilla in breast cancer in 1999 as part of the ALMANAC trial. After the initial trial results in 2004, SLNB was adopted as the standard of care for all patients with clinical or radiological T1 cancers. Axillary clearance without sentinel node biopsy remained the standard approach for those with more advanced tumours. During 2006, the application of SLNB was extended to tumours up to 3 cm in diameter. From December 2007, after the introduction of intra-operative assessment using a PCR-based assay, SLNB became the index standard for axillary assessment of all breast cancer patients irrespective of T-stage, unless there was clinical or radiological evidence of nodal involvement. All operations were performed or supervised by consultant surgeons, unless a dedicated breast surgical trainee had completed accreditation with the SLNB technique. Sentinel nodes were sectioned into 2 mm slices and alternate sections analysed by PCR and conventional histology.

### 2.2. Patient Cohorts

Portsmouth Breast Unit maintains a prospective database of all patients undergoing surgical treatment of breast cancer. From this, 50 consecutive histologically node-positive patients were retrospectively identified in each of the three groups. The sample size was determined by the size of our smallest group (those undergoing iALND), with the other patient groups matched in number. Group 1 consisted of consecutive node-positive patients who had undergone primary axillary clearance prior to November 2007; group 2 consisted of patients who had undergone SLNB and proceeded to dALND following histological node analysis following the introduction of SLNB but prior to the adoption of intra-operative analysis (between November 2007 and November 2009); group 3 consisted of consecutive patients who had undergone SLNB and iALND following positive intra-operative SLNB PCR analysis (after November 2009).

### 2.3. Data Collection

Data was collected from electronic histopathological and clinic letter databases and analysed using SPSS v14 (SPSS Inc., IBM). Data collected included age, sex, tumour type, grade, size (T score), hormone receptor status (oestrogen and progesterone), HER-2 receptor status, and the nature of surgery performed on the breast primary. Histological invasive tumour size was documented in all cases, and total tumour size was noted in the histology report. T score was determined on the basis of invasive tumour size or multifocality. Statistical analysis was performed to test the null hypothesis that all patients were taken from the same population group. Age was analysed using analysis of variance (ANOVA), and all other parameters were assessed using Pearson's Chi-Squared test.

Outcome measures were determined. The primary outcome was the total number of lymph nodes harvested from the axilla (combining axillary dissection with sentinel node harvest where performed). The secondary outcome was the total number of histologically positive lymph nodes harvested. Where not explicitly stated, nodal metastases were assumed to be macrometastases without extracapsular spread. Where lymph nodes were PCR positive but histologically negative, histological specimens were sent for immunohistochemistry. Statistical significance testing against the null hypothesis was performed using ANOVA.

## 3. Results

Our three groups were statistically similar in all respects, except the nature of surgery performed on the primary breast tumour (see Table 1). Our patient groups were similar in terms of sex, with only one male among 150 patients. The mean ages ranged from 59.6 to 63.4 years. Tumour size, as reflected by T score, was similar across patient groups, with the majority of tumours less than 5 cm in diameter (T1 and T2). There were similar numbers of multifocal tumours in the three groups (range 7 to 11). Tumour grades were similar, with the majority of tumours being graded 2 or 3, and most tumours were of ductal type. Oestrogen and progesterone receptors positivity dominated, and few tumours tested positive for HER-2 receptors. Patients in group 1 were more likely to have their primary tumours treated with mastectomy than breast conserving treatment, in contrast with groups 2 and 3. This difference was most marked in group 2, where only 2 patients underwent mastectomy. These differences in nature of surgery performed on the breast were strongly statistically significant (*P* < 0.001).

**Figure 1:**
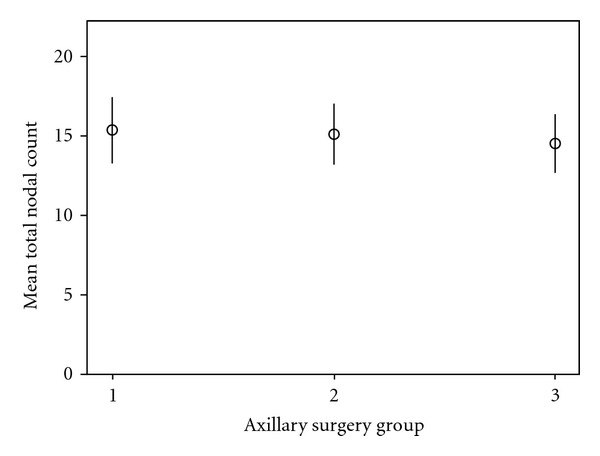
Mean total number of nodes harvested in the three cohorts (±95% confidence interval). Group classification of patients by axillary procedure: group 1: rimary axillary clearance; group 2: SLNB and dALND; group 3: SLNB and iALND.

**Figure 2:**
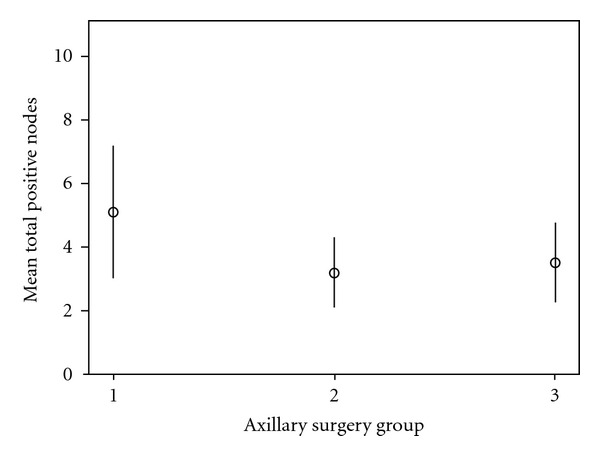
Mean number of positive nodes harvested in the three cohorts (±95% confidence interval). Group classification of patients by axillary procedure: group 1: primary axillary clearance; group 2: SLNB and dALND; group 3: SLNB and iALND.

All three patient groups were statistically similar regarding both outcome measures—total number of nodes harvested from the axilla and the total number of positive nodes harvested (Table 2 and Figures 1 and 2). The mean total number of nodes harvested ranged from 14.6 to 15.4 with clearly overlapping 95% confidence intervals as illustrated in Figure 1. The mean number of positive nodes was higher in group 1 at 5.1, compared with 3.2 and 3.52 in groups 2 and 3, respectively, but 95% confidence intervals overlap, and no statistically significant difference was found (Figure 2). Consistent with this the median total number of nodes harvested was 14 in all three groups. The median number of positive nodes was also 2 across all patient groups.

## 4. Discussion

Here, we demonstrate that both the total number of lymph nodes harvested from the axilla and the number of positive nodes are unaffected, whether axillary clearance is performed as a primary procedure, as a delayed procedure following SLNB, or as an immediate procedure following intra-operative analysis of sentinel lymph nodes (SLN). This confirms recent findings [8].

The mean number of positive nodes harvested was higher, although not significantly so, in patients undergoing primary axillary clearance compared to those having an axillary clearance following SLNB. This tendency may reflect bias due to preoperative selection of patients with clinically or radiologically positive nodes in the primary axillary clearance cohort.

Our patient groups were comparable over all parameters except the nature of surgery performed on the primary breast tumour. This demonstrates that our results are valid, as although our patients were selected by being consecutive rather than through a rigorous case matching process, the groups were statistically similar in all but one measured characteristic. The difference between groups in the nature of surgery performed is likely to be the result of previous local policy, prior to the introduction of intra-operative SLN analysis, if only offering SLNB to patients with smaller breast tumours undergoing breast conserving surgery. With the introduction of intra-operative SLN analysis, SLNB was offered to all clinically and radiologically node-negative patients.

It is documented that nodal harvest is determined not only by surgical technique, but also by the degree to which nodes are pursued at histopathological analysis [10]. In this single-centre study, we believe that variations in lymph node count due to individual surgeon or pathologist-specific technique is likely to have been minimised by standardised surgical and pathological protocols.

## 5. Conclusion

We conclude from this study that there is no statistically significant difference in the number of lymph nodes or number of positive nodes harvested from the axilla, regardless of timing of axillary clearance or the use of SLNB. The goal of axillary clearance is to remove all potentially involved lymph nodes and fatty tissue, and using the number of nodes as a measure of adequacy of tissue retrieval, we have shown equivalence between each technique in our unit. Therefore, even in more surgically challenging circumstances, such as the scarred axilla of a previous SLNB, surgical technique is robust in ensuring adequate clearance of the axilla.

## Figures and Tables

**Table 1:** Clinicopathological Features. Clinico-pathological features for the three cohorts examined. Age was analysed using Analysis of Variance (ANOVA), other parameters using Pearson's Chi-Squared test. Group classification of patients by axillary procedure: group 1: primary axillary clearance; group 2: SLNB and dALND; group 3: SLNB and iALND.

		Group 1	Group 2	Group 3	Significance (*P*)
Sex	F : M	50 : 0	49 : 1	50 : 0	0.365
Age	Mean	63.4	59.6	59.6	0.191^a^
	95% CI	59.2–67.5	56.6–62.6	56.8–62.4	

T size	1	16	28	13	0.096
	2	21	15	21	
	3	3	0	4	
	4	1	0	1	
	MF	9	7	11	

Grade	1	3	5	5	0.728
	2	27	29	32	
	3	19	16	12	
	Other	1	0	1	

Type	Ductal	41	40	39	0.957
	Lobular	3	4	3	
	Other	6	6	8	

ER	−ve	14	7	10	0.222
	+ve	36	43	40	

PR	−ve	16	9	12	0.265
	+ve	34	41	38	

HER-2	−ve	37	40	35	0.521
	+ve	3	5	4	
	Indeterminate	10	5	11	

Surgery	Local excision	16	48	29	<0.001
	Mastectomy	30	2	13	
	Oncoplastic	4	0	8	

^a^Analysis of Variance.

**Table 2:** Outcome Measures. Group classification of patients by axillary procedure: group 1: primary axillary clearance; group 2: SLNB and dALND; group 3: SLNB and iALND.

		Group 1	Group 2	Group 3	Significance (*P*)
Total nodes	Median	14	14	14	0.822
Positive nodes	Median	2	2	2	0.157
